# Enhancing Emergency Preparedness: A Call to Incorporate Postexposure Prophylaxis in Mass Casualty Protocols

**DOI:** 10.1002/hsr2.70973

**Published:** 2025-06-23

**Authors:** Majani Edward, Archie Hellar, Chizaram Onyeaghala, Oluruntoba Ogunfolaji

**Affiliations:** ^1^ Department of Public Health St. Francis University College of Health and Allied Sciences Ifakara Tanzania; ^2^ University of Port Harcourt Hospital Port Harcourt Nigeria; ^3^ Research Department Association of Future African Neurosurgeons Yaounde Cameroon

**Keywords:** HIV, mass casualty, PEP

## Abstract

**Introduction:**

Mass casualty incidents (MCIs) present significant challenges to emergency response systems, often overwhelming healthcare resources. This article highlights the critical role of integrating postexposure prophylaxis (PEP) into emergency protocols to mitigate immediate and secondary health risks occurring due to mass casualty. The aim is to underscore the importance of PEP in MCI management and advocate for its systematic incorporation into emergency preparedness and response.

**Methods:**

This is a perspective article drawing upon existing literature, guidelines from organizations such as UNAIDS, and expert opinion to synthesize the importance of PEP in the context of MCIs. It discusses the rationale for PEP, challenges in its implementation, particularly in resource‐limited settings, and proposes strategies for its integration into emergency protocols.

**Results:**

The analysis underscores that prompt PEP administration significantly reduces the risk of bloodborne pathogen transmission following potential exposure during MCIs. Effective MCI response necessitates hospital preparedness, robust Emergency Medical Services (EMS), interagency collaboration, and trained personnel. The potential for HIV transmission in crash emergencies is often overlooked, highlighting the urgent need for PEP inclusion in emergency protocols. Key recommendations include establishing robust surveillance systems, training first responders, ensuring the availability of personal protective equipment and point‐of‐care test kits, establishing blood bank networks, and ensuring widespread access to PEP with a low threshold for its administration.

**Conclusion:**

Integrating PEP into emergency response strategies is imperative for enhancing the resilience and preparedness of healthcare systems, especially in resource‐constrained settings. This proactive approach can significantly reduce morbidity, mortality, and the public health burden associated with infectious disease transmission following MCIs. Therefore, the systematic incorporation of PEP into emergency protocols, supported by interagency collaboration, training, and resource allocation, is strongly recommended.

## 1 Introduction

1.1

Mass casualty incidents (MCIs) pose a significant challenge to emergency response systems [1, 2]. These events overwhelm healthcare resources and require swift, coordinated action to minimize casualties [3]. Effective preventive measures and protocols are crucial for mitigating the immediate health risks associated with MCIs. Postexposure prophylaxis (PEP) emerges as a critical element in preventing secondary health issues following MCIs [4]. Exposure to bloodborne pathogens like Hepatitis B and C viruses (HBV and HCV) poses a significant threat to healthcare workers and casualties alike [5]. PEP medications can significantly reduce the risk of contracting these infections when administered promptly after potential exposure [6]. Hospital preparedness is a key factor in ensuring effective MCI response [7]. Hospital preparedness, encompassing adequate surge capacity with sufficient beds, ambulances, and trained personnel, forms the bedrock of an effective MCI response. Similarly, efficient Emergency Medical Services (EMS) are vital in the immediate aftermath of an MCI, and continuous efforts to enhance their effectiveness are paramount [7, 8]. This perspective article underscores the paramount significance of integrating PEP into emergency protocols for MCI management. It calls for proactive measures to prevent secondary health issues stemming from exposure to bloodborne pathogens, particularly in the context of road traffic accidents and other crash emergencies where such risks may be inadvertently overlooked amidst the urgency of acute care.

## 2 Significance of Postexposure Prophylaxis in Preventing Secondary Health Issues

PEP is a crucial intervention, highly effective in preventing disease development and, in some instances, minimizing secondary transmission to other susceptible persons [6]. PEP is routinely recommended following exposure to a wide spectrum of viral and bacterial diseases [4]. Thus, physicians should be familiar with established PEP protocols. Exposure to communicable diseases occurs in occupational and nonoccupational settings [6]. The infectious agent can be transmitted by another person, an animal, or the environment [9]. The primary goals of a physician's evaluation after an exposure are to provide PEP if indicated, to allay the exposed person's anxiety, and to avoid unnecessary interventions and loss of workdays [7]. PEP can be applied in the following infectious pathogens; human immunodeficiency virus (HIV), hepatitis B virus, hepatitis A virus, varicella zoster virus, influenza and rabies viruses [10]. It can also be applied for bacterial infections such as invasive group A streptococcal infection, invasive meningococcal infection, pertussis, tetanus, and tuberculosis [8].

HIV PEP aims to reduce the risk of HIV transmission through the use of antiretroviral medications [11]. PEP should be prescribed after occupational exposure to HIV by health workers and following nonoccupational exposures, including unprotected sexual exposure, injecting drug use, and exposure following sexual assault [6]. To achieve its effect of blocking viral replication and preventing transmission, PEP should be given within 2 h (ideally) and no later than 72 h following exposure. The WHO recommends at least a two‐drug PEP regimen, but prefers three drugs. The efficacy of PEP in reducing the risk of HIV transmission has been reported to be in excess of 80% [10]. Patients with potential HIV exposures, both occupational and nonoccupational in nature often present to the Emergency Department (ED) due to its ability to provide round‐the‐clock time‐sensitive evaluation and management of body fluid exposures [11]. As such, it is imperative that attending ED physicians be familiar with the nature of presentation of such patients to the ED and the guidelines used for performing a risk assessment to determine the need for PEP in these patients [11]. Commencement of a full 28‐day prescription of PEP is time‐sensitive and requires risk assessment that considers the source patient's risk profile and the nature of exposure including the infectious material involved and enhanced adherence counseling [4]. PEP plays a huge role in preventing the onset of secondary health issues such as infectious diseases (HIV, hepatitis B and C, tuberculosis, and anthrax) and psychological trauma, which may not be immediately apparent being that a traumatic event with an identifiable point of onset [12]. Therefore, PEP can be viewed as a form of secondary prevention in asymptomatic high‐risk patients with implication for improving the overall well‐being of the population.

## 3 Call to Action for Incorporating Postexposure Prophylaxis Into Emergency Protocols

The impact of HIV transmission in crash victims has often been ignored by the majority of stakeholders and first responders as compared to others in the general population [13]. This is likely due to the fact that crash victims often have life‐threatening injuries which are the focus of the acute evaluation of emergency responders [14]. Also, while HIV can have long‐lasting impact and consequences on individual and public health, it is often not seen as a major health challenge in the acute phase as other injuries may seem [13]. In the care of crash victims, especially those with multiple trauma, life‐saving interventions are very often instituted, either at the site of the crash, or in the hospital, often requiring interventions such as emergency surgeries and blood transfusions, sometimes on multiple patients [14]. This raises the risk of transmission of infectious agents among such victims [15]. This is increased in populations with a high prevalence of HIV as sometimes, adequate triage and screening of victims may not be feasible during the acute care phase [16]. A lot of African countries fall within the lower‐middle Income and low‐income classification of countries [17] and as such, often cannot accrue enough resources for standard screening of emergency victims in the acute stage of their care.

This problem as elucidated leads to the question of what should be done for HIV in crash emergencies? The Joint United Programme on HIV and AIDS (UNAIDS) released guidelines for basic prevention of HIV in emergencies and emergency‐affected populations in 1996 [15], however, humanitarian actors have not fully latched on to the basics of the guidelines especially in Africa. While the guidelines relate more to emergency‐affected populations such as war‐torn areas, and areas affected by massive displacement of people due to natural disasters, it also has importance in the prevention of HIV transmission as it relates to crash emergencies [10]. There is a need for interagency collaboration in the prevention and care of individuals involved in road traffic accidents and crash injuries/emergencies, as local authorities and health institutions alone in lower‐middle‐income and low‐income countries may not be able to mount appropriate resources in the acute management phase of such victims [16]. The development of foolproof surveillance systems in capturing data of HIV incidence, prevalence and care needs to be vital in the overall institution of prophylactic and long‐term care of individuals in emergency situations as this helps to map out transmission density thus informing policies and decisions regarding the establishment of urgent care protocols in the prevention of HIV [15, 16]. Training of first responders and emergency care personnel is paramount in the prevention of HIV transmission between victims involved in crash emergencies, with the provision and use of adequate and standard personal protective equipment, point‐of‐care test kits and adequate triage of victims in such emergency situations [4]. The establishment of a network of blood banks or blood donation service stations in densely populated and emergency‐prone areas is needed to ensure the transfusion of adequately screened and safe blood and blood products in cases of mass transfusion [9]. Lastly, the importance of widely available access to PEP in suspected cases of inadvertent blood–blood exposure or victim–victim exposure during or after the acute on‐site/in‐hospital care cannot be overemphasized [9]. Emergency and clinical staff should have a low threshold of suspicion of possible exposure in such situations to adequately step‐up prophylactic care, thus preventing and minimalizing stigmatization, further spread of disease, increase of morbidity and mortality and public health spending on HIV care in resource‐constrained settings [16].

## 4 Conclusion and Recommendation

MCIs present a substantial challenge to emergency response systems, particularly in regions with limited resources. The systematic implementation of effective preventive measures and protocols is essential to mitigate both the immediate and secondary health risks associated with these events. PEP stands out as a critical intervention to prevent secondary health issues arising from exposure to bloodborne pathogens, such as HIV, hepatitis B, and C. PEP is crucial for healthcare workers and casualties in both occupational and nonoccupational settings, significantly reducing the risk of contracting infections when administered promptly. The efficacy of PEP, especially in preventing HIV transmission, underscores the need for EDs and healthcare providers to be well‐versed in PEP protocols and risk assessments.

To enhance the overall effectiveness of MCI responses, it is imperative that hospitals maintain adequate surge capacity, including sufficient beds, ambulances, and trained personnel. EMS play a vital role in the immediate response, and ongoing efforts to improve their effectiveness are crucial. There is an urgent and compelling need to formally incorporate PEP into standard emergency protocols, particularly in crash emergencies, where the risk of transmission of infectious agents is heightened. This integration necessitates the establishment of robust surveillance systems to monitor potential exposures, comprehensive training for all first responders and emergency personnel on PEP guidelines, and ensuring the consistent availability of necessary resources such as personal protective equipment and point‐of‐care test kits. Moreover, the development of well‐supported blood bank networks and ensuring widespread and readily accessible PEP for all suspected exposures are critical components of a comprehensive strategy. By proactively addressing the potential for secondary health issues through the incorporation of PEP into emergency protocols, we can significantly improve the overall well‐being of populations affected by MCIs. This proactive approach not only reduces morbidity and mortality associated with preventable infectious but also minimizes the long‐term public health burden and economic impact, particularly in resource‐constrained settings. Therefore, the strategic and comprehensive integration of PEP into emergency response frameworks is an imperative step toward enhancing the resilience and preparedness of healthcare systems globally.

## Author Contributions

**Majani Edward:** conceptualization, writing – original draft, writing – review and editing, supervision, investigation, formal analysis. **Archie Hellar:** conceptualization, writing – original draft, investigation. **Chizaram Onyeaghala:** writing – original draft. **Oluruntoba Ogunfolaji:** writing – original draft.

## Ethics Statement

The authors have nothing to report.

## Consent

The authors have nothing to report.

## Conflicts of Interest

The authors declare no conflicts of interest.

## Transparency Statement

The lead author Majani Edward affirms that this manuscript is an honest, accurate, and transparent account of the study being reported; that no important aspects of the study have been omitted; and that any discrepancies from the study as planned (and, if relevant, registered) have been explained.

## Data Availability

All data were retrieved from the academic search. The data that support the findings of this study are available from the corresponding author upon reasonable request.
